# Novel association between FOXO3 rs2232365 polymorphism and late-onset preeclampsia: a case-control candidate genetic study

**DOI:** 10.1186/s12884-020-03479-6

**Published:** 2020-12-14

**Authors:** Xuefeng Pan, Benjie Wei, Hong Wang, Lingyu Ma, Zhaoli Du, Ying Chen

**Affiliations:** 1 Department of Obstetrics, The First Hospital of Jilin University, Xinmin Street 1, Changchun, Jilin Province 130021 China; 2 Institute of Genetic Technology, Yinfeng Bilogical Group, No. Three Road No. 1109, Shandong, Ji’nan Hi Tech Development Zone Export Processing Zone, Jinan, Shandong Province 250014 China

**Keywords:** Preeclampsia, Single nucleotide polymorphism, rs2232365, BMI, Lipid metabolism

## Abstract

**Background:**

Both genetic susceptibility and dysregulated lipid metabolism are important susceptibilities to preeclampsia. In the study, we devote to investigate the associations of FOXO3 and TLR7 genetic polymorphisms with preeclampsia in a Chinese population.

**Methods:**

This case-control study involved 335 Han Chinese pregnant women, including 177 pregnant women with preeclampsia and 158 healthy controls. The preeclampsia group was further sub-grouped into early-onset preeclampsia (EOPE, *n* = 70)and late-onset preeclampsia (LOPE, *n* = 107. Three single nucleotide polymorphisms (SNPs), including FOXO3 (rs2232365, rs3761548), and TLR7 rs3853839 were genotyped by multiplex PCR for targeted next-generation sequencing. The χ^2^ test and multiple interaction effect analyses were performed to determine the association of three SNPs with serum lipid levels and thyroid function in women with preeclampsia.

**Results:**

The genotype (CC vs. TT + CT) distribution of rs2232365 revealed a significant association with LOPE (*P* = 0.004, odds ratio = 3.525 (0.95 CI: 1.498–8.164)). No significant difference was found in the genotype and allele frequencies of rs3761548 and rs3853839 between controls and cases (*P* > 0.05). Moreover, the genotype CT/TT of rs2232365 was significantly correlated with increased TG/HDL levels in the LOPE group (*p* = 0.014).

**Conclusions:**

The polymorphisms of rs2232365 are associated with the risk of LOPE and may modulate TG/HDL levels in pregnant women with LOPE.

## Introduction

Preeclampsia (PE) is a pregnancy-specific syndrome and associated with significant maternal and fetal morbidity and mortality [1]. PE averagely affects 6.7% of pregnant women globally and 4.2% individuals in China [2, 3]. The pathogenesis of PE is multifactorial, with acknowledged contributions by genetic susceptibility, inflammatory stimuli, metabolic syndrome, oxidative stress, placental, and vascular dysfunction [4]. According to epidemiological studies, there was a high prevalence of metabolic syndrome in women with PE [5], otherwise autoimmune diseases and dysregulated lipid metabolism showed a tight association [6]. One of these most important hypotheses of PE is that maternal adequate immunological response is necessary to the existence of the fetus in pregnancy [7].

Forkhead/winged helix transcription factor(FoxP3)is the key transcription factor for Regulatory T cells (Tregs) differentiation and function [8]. Tregs, as a specialized subset of immune cells, plays an important role in the establishment and maintenance of immune tolerance [9]. FoxP3 is responsible for the differentiation of Tregs to a suppressive phenotype and stabilizing their lineage [10]. There are several conserved noncoding sequences, designated as CNS 0–3, in the genomic region of Foxp3 locus. CNS 0–3 holds different signaling pathways respectively and deficiency of FoxP3 will impair the suppressive activity of Tregs [11]. A meta-analysis study showed that forkhead box protein 3 polymorphisms (rs2232365, rs3761548) were associated with the outcome of immune-related pregnancy complications. Immunological incompatibility between mother and fetus is frequently observed in preeclampsia and genetic factors related to the immunological pathway in preeclampsia have been discovered [12]. In Asian, rs3761548 polymorphism was significantly associated with multiple sclerosis, an immune-related central nervous disease [13]. In the Chinese Han population, rs2232365 and rs3761548 polymorphisms confer an important susceptibility to unexplained recurrent spontaneous abortion by altering Foxp3 function and/or its expression [14].

Toll-like receptors(TLRs)is critical innate immune activators, which can affect Treg-dependent immune regulation by reducing the number of Tregs [15, 16]. TLRs, as innate immunity sensors, play important roles in the activation of innate and adaptive immune responses [17]. Polymorphisms of TLR7 rs3853839 are associated with the susceptibility to Chikungunya virus (CHIKV) infection in Indian people and the severity of EV71 in Chinese boys [18, 19].

Although the precise mechanism of preeclampsia is not understood, the disease is thought to occur as soon as the placenta was implanted [20]. Delay in childbearing, obesity, metabolic disorders, and genetic risk are all involved in this development of preeclampsia [21]. This study aimed to investigate whether rs3853839, rs2232365, and rs3761548 located in the X-chromosome are associated with preeclampsia in a Chinese case-control cohort, with a specific focus on the biochemical metabolic parameters.

## Material and methods

### Study subjects

**Fig. 1:**
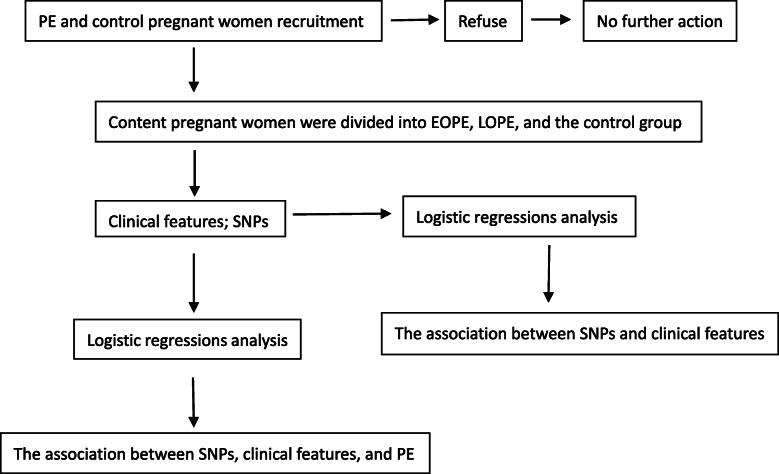
Flow chart showing the steps in participant analysis

The study included 335 individuals who provided informed written consent, including 177 pregnant women with preeclampsia and 158 healthy controls. The patient group was further classified into two subsets: 70 patients with early-onset preeclampsia (EOPE, defined as preeclampsia diagnosed within 34 + 0 weeks of gestation) and 107 patients with late-onset preeclampsia (LOPE, defined as preeclampsia diagnosed after 34 + 0 weeks of gestation) following the latest ACOG Practice Bulletin No. 202: Gestational Hypertension and Preeclampsia [22]. The diagnostic criteria for PE is defined as blood pressure ≥ 140/90 mmHg after 20 weeks of gestation. Severe hypertension is defined as blood pressure ≥ 160/110 mmHg with or without proteinuria, accompanying the following symptoms: liver injury, renal insufficiency, pulmonary edema, cerebral or visual disturbance. This research project was performed in accordance with the Declaration of Helsinki, and ethical approval was obtained from the local Ethics Committee of the First Hospital of Jilin University, Changchun, China (Permission number: 2018 − 401). All individuals were from the Han population in Northeast China and signed a written informed consent form. Figure 1 shows a chart of the trial design.

Exclusion criteria included patients with diabetes mellitus, chronic hypertension, diseases of the blood system, cardiovascular diseases, renal disease, and cerebrovascular accidents. Pregnant women exhibiting elevated blood pressure without proteinuria were also excluded from the study. All the controls were confirmed with normal blood pressure and without any other chronic diseases.

### Patient and public involvement

This case-control study was conducted between May 2018 and January 2020 in the department of Obstetrics of the First Hospital of Jilin University in China. Using logistic regression, our study involved 15 explanatory variables, and since the minimum observations required are multiplied by 10 to yield significant results, our studied sample size was determined as above 300 cases. The design of this study was not directly involved in the diagnosis and treatment of patients, and the intervention is not considered to affect the patient’s therapy. Patients in hospital were recruited voluntarily when they received medical history collection. The meaningful results for therapeutic effect were disseminated to study participants, such as lipid metabolism and thyroid function, and uncertain genetic information were reserved. In the study, no additional burden were added to those patients themselves for the funding supported the Jilin Province Science Development Plan.

### Measures

Gestational age was calculated by the last menstrual period and the first ultrasound in the first trimester. Systolic blood pressure (SBP), diastolic blood pressure (DBP), height, and weight of each patient were measured before treatment according to the Seventh Report of the Joint National Committee on Prevention, Detection, Evaluation, and Treatment of High Blood Pressure [23]. Venous blood samples (3 mL) from every participant were obtained from the subjects after a 12-hour fast. The circulation levels of thyrotropin, thyroid-stimulating hormone(TSH), triiodothyronine (T3), thyroxine(T4), triglyceride(TG), total cholesterol(TC), high-density lipoprotein cholesterol (HDL-C), and low-density lipoprotein cholesterol (LDL-C) were measured by an automatic biochemical analyzer (SEKISUI medical technology Ltd., Tokyo, Japan).

### DNA extraction and genotyping

Genomic DNA was extracted from 3 mL EDTC-anticoagulated blood samples using a Gene JET Whole Blood Genomic DNA Purification kit (Thermo Scientific Co. Ltd.). SNPs were analyzed through multiplex PCR for targeted next-generation sequencing.

### Statistical analysis

All statistical analyses were performed using Microsoft Excel (2007) and IBM SPSS Statistics for Windows (version 18.0, IBM Corporation., Armonk, NY, USA). Genotype numbers were determined by manually counting and allele frequency was determined from the genotype frequencies. The calculation for Hardy-Weinberg equilibrium was performed and genotype distribution was determined. (available at http://ihg.gsf.de/cgi-bin/hw/hwa1.pl). Normality distributed continuous variables were presented as mean ± standard deviation (SD) for parametric variables, and continuous variables with non-normal distribution are represented by the median (Q1-Q3). Non-normal distribution data were compared between the studied groups using U-test. The chi-square test was used to assess the associations of genotypes and alleles with preeclampsia. Haplotype analysis was predicted from genotype data by the computer program Haploview. Binary logistic regression analysis was also performed to identify independent risks of preeclampsia and linear regression analysis was used for the risk of high TG/HDL in LOPE. The odds ratios (ORs) and 95% confidence intervals (CIs) were calculated, and *P* < 0.05 for all pairwise comparisons; *P* < 0.017 for the multiple comparisons, according to Bonferroni correction were considered significant.

## Results

### Clinical characteristics

The general characteristics of all participants, including controls, EOPE, and LOPE, were presented in Table 1. None of them was missing and all blood samples were tested successfully. There were significant differences in multiple parameters between the PE group and the control group, but the differences were not consistent. Pregnant women with EOPE had higher levels of BMI, TSH, FT3 and TG/HDL than controls. In the LOPE subgroup, weight, BMI, TSH, FT3, TG, TG/HDL were different from those of the control group. *P* < 0.05 for all pairwise comparisons; *P* < 0.017 for the multiple comparisons, according to Bonferroni correction.

**Table 1:** Demographic and clinical features of the study subjects

Variables	Controls(*N* = 158)	PE (*n* = 177)	EOPE (*n* = 70)	LOPE (*n* = 107)	*P*^a^ Value	*P*^b^ Value	*P*^c^ Value
Maternal age ( years)	30.00(28.00–33.00)	31.00(28.00–35.00)	33.00(28.00–37.00)	30.00(27.00-33.50)	0.078	0.001*	0.935
Gestation at delivery (weeks)	39.43(38.86–39.86)	35.43(32.57–37.57)	31.86(29.86–33.14)	37.00(35.93–38.43)	< 0.001 *	< 0.001 *	< 0.001 *
Height	162.50(160.00-165.00)	162.00(159.00-165.00)	160.00 158.00-164.00)	162.50(159.50–165.00)	0.648	0.042	0.388
Weight	71.00 (66.00–80.00)	76.00 (70.00–87.00)	75.00(70.00-82.50)	78.00(70.00-89.50)	< 0.001 *	0.017	< 0.001 *
BMI	27.34(25.00-29.655)	29.14(26.80–32.80)	28.76(26.71–32.23)	29.30(26.89–33.10)	< 0.001 *	0.001*	< 0.001 *
TSH(Ulu/mL)	2.06(1.51–2.89)	3.69(2.29–5.19)	3.66(2.21–4.87)	3.69(2.35–5.31)	< 0.001 *	< 0.001 *	< 0.001 *
FT3	4.75(4.17–5.19)	3.91(3.40–4.43)	3.72(3.31–4.21)	4.01(3.45–4.53)	< 0.001 *	< 0.001 *	< 0.001 *
FT4	10.94(9.84–13.13)	11.82(10.37–13.45)	11.32(10.37–13.45)	12.02 (10.59–13.54)	0.019*	0.496	0.004*
TC (mmol/L)	6.06 (5.36–6.95)	6.03 (5.13–7.06)	6.14 (5.27–7.48)	5.98 (5.07-7.00)	0.186	0.806	0.186
TG (mmol/L)	3.05 (2.46–3.96)	3.51 (2.80–4.51)	3.48 (2.65–4.43)	3.57 (2.84–4.54)	0.002 *	0.026	0.002 *
HDL-C (mmol/L)	1.86 (1.65–2.11)	1.71 (1.44–2.05)	1.76(1.49–2.11)	1.68(1.39–1.98)	< 0.001 *	0.128	0.001 *
LDL-C (mmol/L)	2.87 (2.42–3.48)	3.02 (2.52–3.72)	3.10 (2.62–3.87)	2.92 (2.40–3.70)	0.460	0.052	0.532
TG/HDL-C	1.74 (1.32–2.24)	2.14 (1.54–2.81)	2.08 (1.52–2.65)	2.19 (1.57–2.90)	< 0.001 *	0.006*	< 0.001 *

*PE* represents Preeclampsia, *EOPE* represents early- onset preeclampsia, *LOPE* represents late-onset preeclampsia, *BMI *Body mass index, *TSH *Thyroid-stimulating hormone, *FT3 *Free triiodothyronine, *FT4 *Free thyroxine, *TC *Total cholesterol, *TG *Triglyceride, *HDL-C *High-density lipoprotein, *LDL-C *Low-density lipoprotein, *TG/HDL *Triglyceride/ high-density lipoprotein

All the continuous variables were presented as median and the 25th-75th percentile for non-normal distribution tested. *P* values were analysis using U-test

^a^ PE vs. controls, ^b^ EOPE vs. controls, ^c^ LOPE vs. controls, *P*^a^ < 0.05; * *P*^b^ and *P*^C^ < 0.017 for sub-group

### Genotype and allele frequencies of cases and controls

Genotype frequencies of cases and controls are shown in Table 2. Hardy-Weinberg equilibrium was tested in the control group, and the result was consistent with the expectation. There was no significant difference of other genotypic frequencies between women with PE or EOPE and controls. Although the variants of rs2232365 and rs3853839 showed no significant association with LOPE risk (*P*>0.017), which was also brought into binary logistic regressions to analyze the association between LOPE and different parameters.

**Table 2:** Genotype and allele frequencies between study groups

SNP ID	Model	Controls *N* = 158 (100%)	PE			EOPE			LOPE		
SNPs			*N* = 177(%)	ϰ^2^	*P*^a^	*N* = 70(%)	ϰ^2^	*P*^b^	*N* = 107(%)	ϰ^2^	*P*^C^
rs2232365	CC	21(13.29%)	35(19.77%)	4.016	0.134	10(14.29%)	0.799	0.671	25(23.36%)	5.682	0.058
	CT	80(50.63%)	93(52.545)			39(55.71%)			54(50.47%)		
	TT	57(36.08%)	49(27.68%)			21(30.00%)			28(26.17%)		
Genotype	TT+CT/CC	137(86.71%)	142(80.23%)	2.520	0.112	60(85.71%)	0.041	0.840	82(76.63%)	4.513	0.034
	CC	21(13.29)	35(19.77%)			10(14.29%)			25(23.36%)		
Allele	C	122(38.61%)	163(47.25%)		0.052	59(42.14%)		0.477	104(48.60%)		0.021
	T	194(61.39%)	191(55.36%)			81(57.86%)			110(51.40%)		
rs3853839	CC	10(6.33%)	15(8.47%)	3.731	0.155	7(10.00%)	0.949	0.622	8(7.48%)	6.490	0.039
	CG	78(49.37%)	69(38.98%)			33(47.14%)			36(33.64%)		
	GG	70(44.30%)	93(52.54%)			30(42.86%)			63(58.87%)		
Genotype	CC+CG	88(55.70%)	84(47.46%)	2.268	0.132	40(57.14%)	0.041	0.839	44(41.12%)	5.412	0.020
	GG	70(44.30%)	93(52.54%)			30(42.86%)			63(58.88%)		
Allele	C	98(31.01%)	99(28.70%)		0.747	47(33.57%)		0.293	52(24.30%)		0.092
	G	218(68.99%)	255(73.91%)			93(66.43%)			162(75.70%)		
rs3761548	GG	88(55.70%)	87(49.15%)	1.489	0.475	33(47.14%)	1.534	0.464	54(50.47%)	0.713	0.700
	GT	63(39.87%)	82(46.33%)			34(48.57%)			48(44.86%)		
	TT	7(4.43%)	8(4.52%)			3(4.29%)			5(4.67%)		
Genotype	TT+TG	88(55.70%)	87(49.15%)	1.433	0.231	33(47.14%)	1.425	0.233	54(50.47%)	0.701	0.401
	GG	70(44.30%)	90(50.85%)			37(52.86%)			53(49.53%)		
Allele	G	239(75.63%)	256(74.20%)		0.329	100(71.43%)		0.343	156()72.90%		0.478
	T	77(24.37%)	98(28.41%)			40(28.57%)			58(27.11%)		

*PE* Represents Preeclampsia, *EOPE* Represents early- onset preeclampsia, *LOPE* Represents late-onset preeclampsia. ^a^ PE *vs* controls, ^b^ EOPE *vs* controls, ^c^ LOPE *vs* controls, * *P*^a^ < 0.05; * *P*^b^ and *P*^C^ < 0.017 for sub-group

There was not a significant association between the C allele of rs2232365 and LOPE risk in Chinese Han pregnant women (*P* = 0.021), but binary logistic regressions still were calculated. Furthermore, similar associations were also observed in EOPE groups. We did not find any relationships between alleles of SNPs rs3853839 and rs3761548 and PE.

### Association between genotype variants and clinical and biochemical parameters

Table 3 depicted the laboratory parameters according to the significant SNPs in LOPE individuals. LOPE patients with CC genotypes of rs2232365 showed significantly higher levels of HDL and lower TG/HDL compared to TT + CT genotypes. Furthermore, significantly lower levels of T3 were found in the GG genotype of rs3853839 between LOPE patients as compared to the controls (*P* = 0.005), while the parameter showed no association in the analysis of linear regression. However, no significant differences were observed between laboratory factors and SNPs rs3761548 in LOPE subjects (*P*>0.05 for all comparisons). In EOPE, no significant associations were observed between genotype and biochemical parameters, which was not shown here.

**Table 3:** Association between genotype variants and clinical and biochemical parameters in LOPE groups

SNP ID	Model (n)	BMI	TC	TG	HDL	LDL	TG/HDL	TSH	T3	T4
rs2232365(C/T)	TT + CT(82)	29.01(26.63–34.05)	5.80(5.06–7.06)	3.72(2.81–4.66)	1.61(1.33–1.95)	2.91(2.36–3.72)	2.35(1.64–3.23)	3.29(2.31–5.42)	4.05(3.49–4.48)	12.14(10.59–14.24)
	CC(25)	29.88(28.26–31.96)	6.21(5.22–6.75)	3.30(2.89–3.99)	1.77(1.63–2.10)	2.97(2.46–3.58)	1.88(1.52–2.32)	4.44(3.18–5.19)	3.93(3.45–4.58)	12.00(10.74–12.64)
	*P*	0.219	0.688	0.118	0.040*	0.874	0.016*	0.475	0.953	0.371
rs3853839(C/G)	CC + CG(44)	30.17(26.83–34.18)	5.64(4.67–6.47)	3.51(2.84–4.50)	1.55(1.30–1.86)	2.77(2.38–3.44)	2.32(1.84–2.88)	3.84(2.06–5.75)	4.38(3.86–4.98)	12.08(10.33–13.27)
	GG(63)	29.00(26.91–32.43)	6.21(5.22–7.06)	3.60(2.84–4.59)	1.74(1.49–2.07)	3.02(2.45–3.93)	2.09(1.48–3.03)	3.68(2.62–5.31)	3.87(3.35–4.38)	12.00(10.90-13.64)
	*P*	0.296	0.141	0.648	0.063	0.222	0.425	0.894	0.005*	0.423
rs3761548(T/G)	GG + TG(102)	29.10(26.76–32.77)	5.96(5.06–7.02)	3.57(2.81–4.51)	1.67(1.38–1.97)	2.95(2.39–3.77)	2.18(1.53–2.92)	3.70(2.39–5.43)	4.02(3.45–4.52)	12.08(10.58–13.54)
	TT(5)	34.29(29.24–35.20)	6.57(5.46–6.75)	3.60(3.35–4.96)	1.78(1.62–2.13)	2.77(2.70–3.10)	2.72(1.94–2.79)	3.65(1.96–4.74)	3.98(3.87–4.58)	12.00(11.86–12.02)
	*P*	0.083	0.831	0.456	0.560	0.745	0.626	0.658	0.935	0.859

*LOPE* represents late-onset preeclampsia, *BMI *Body mass index, *TSH *Thyroid-stimulating hormone, *FT3 *Free triiodothyronine, *FT4 *Free thyroxine, *TC *Total cholesterol, *TG *Triglyceride, *HDL-C *High-density lipoprotein, *LDL-C *Low-density lipoprotein, *TG/HDL *Triglyceride/ high-density lipoprotein. *P < 0.05 versus controls

### Association between PE and different parameters

**Fig. 2:**
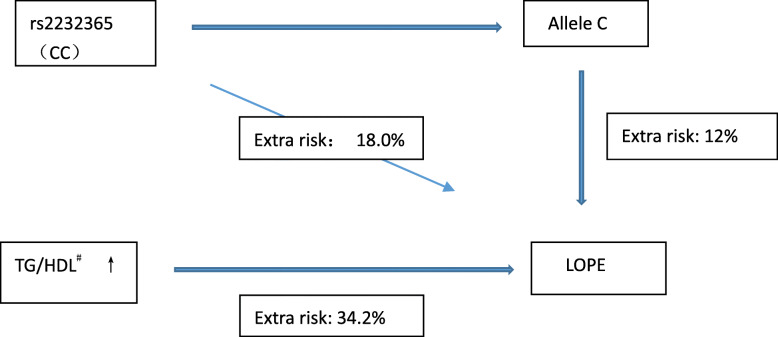
The interactional associations of SNPs and biochemical parameters in LOPE. ^#^The Continuous variable TG/HDL ratio was divided into two groups according to the median (1.843) of all TG/HDL for calculating extra risks

Binary logistic regressions were performed and the results were presented in Table 4. These significant associations appeared after the false-positive discovery amendment. We observed that both SNP rs2232365 (CC) and TG/HDL were associated with LOPE. Linear regressions were further performed to analyze the association between TG/HDL and other parameters, and our study found that SNP rs2232365 related to it based on current data (*p* = 0.027, (0.95 CI: 0.089–1.430)) in those patients with LOPE. To assess exposure risk, extra risk were calculated by converting the odds ratio. The extra risk was 55%, 63.8% and 58.7% higher in the PE, EOPE and LOPE groups, respectively, compared with the control group. We especially assess the absolute risks of CC genotype LOPE, those cases with CC genotype has increasing risk as high as 18% compared with those with CT/TT genotype. The extra risk of TG/HDL was 34.2% in LOPE group (showed in Fig. 2).

**Table 4:** Odds ratios (95% confidence intervals) for the association between PE and different parameters

	Sig.	Odds ratio	95%CI for EXP (B)	
			Lower	Upper
PE				
BMI	<0.001^※^	1.199	1.116	1.289
TG/HDL	<0.001^※^	1.747	1.283	2.379
TSH(uIU)	<0.001^※^	1.982	1.621	2.432
FT4	0.010^※^	1.109	1.025	1.200
EOPE				
BMI	<0.001^※^	1.252	1.127	1.390
TG/HDL	0.014^※^	1.795	1.124	2.878
TSH	<0.001^※^	1.967	1.506	2.569
FT3	<0.001^※^	0.283	0.177	0.450
LOPE				
rs2232365(CC)	0.004^※^	3.511	1.504	8.199
BMI	<0.001^※^	1.248	1.142	1.363
TSH(uIU)	<0.001^※^	2.113	1.646	2.712
FT4	0.003^※^	1.223	1.070	1.396
TG	0.023^※^	0.578	0.360	0.925
TG/HDL	<0.001^※^	4.087	1.938	8.618

^※^ *P* < 0.05 was considered statistically significant. PE represents preeclampsia, EOPE represents late-onset preeclampsia, LOPE represents late-onset preeclampsia, *BMI* Body mass index, *TSH* thyroid-stimulating hormone, *FT3* Free triiodothyronine, *FT4* Free thyroxine, *TG* Triglyceride, *HDL* High-density lipoprotein, *TG/HDL* Triglyceride/ high-density lipoprotein

## Discussion

### Summary of key results

In this study, we tested the correlation of 3 specific X-chromosome-related SNPs with the susceptibility of PE and identified FOXP3 rs2232365 as a novel risk factor of LOPE. Pregnant women with TT/CT genotype had higher TG/DHL in LOPE, which was the first reported and disclosed implicit relationship(Tables 2 and 3 ). We observed the significant associations between LOPE and rs2232365 (CC), BMI, TSH, FT4, TG, and TG/HDL by multiple logistic regressions and TG/HDL ratio was higher in LOPE patients with CC genotype. These findings indicate that we can develop some personalized treatment plans for our patients who were high-risk ones screened by prediction models. For decreasing TG/HDL to reduce the incidence of late-onset preeclampsia, we can enhance diet and exercise management in those women with high-risk factors, especially in those cases with rs2232365 (TT/CT) genotype.

Although multiple studies have investigated mechanisms of PE, and they remain unclear. The balance of immune plays an important role in pregnancy from placentation to delivery. Some papers on oocyte donation (OD) during in vitro fertilization (IVF) cycles show strong evidence that immunity may be involved in PE development. Increased risk of EOLP for pregnant women with oocyte donation (OD) has been observed compared with both spontaneous pregnancies and pregnancies achieved by in vitro fertilization (IVF) with autologous oocytes [24]. The decidua obtained by OD, as a kind of completely allogeneic but partially maternal, showed more genetic and immunological differences and need a more intense downregulation of the maternal alloimmune response [25]. Aberrant human lymphocyte antigen (HLA) allogenicity can alter the function of uterine natural killer cells (uNK) and lead to the abnormal maternal blood supply to the placenta, which is the predominant cause of facilitates disorders such as PE and fetal growth restriction [26]. A recent study also showed abnormally low uterine arteries pulsatility index and serum maternal 17 β -E at 11 to 13 + 6 weeks in oocyte donations as compared to natural conceptions [27, 28]. The authors of this study described this new phenomenon as increasing placental perfusion to compensate for maternal or placental dysfunction [27]. In animal models, the fluctuation of circulating steroids could lead to a reduction in uterine vascular resistance [29]. In addition, a recent study showed that the increased age of a pregnant woman during egg donation increases the risk of PE [29]. The above discoveries seem to explain the hypothesis that older pregnant women whose lower 17 β –E affect the uterine arteries pulsatility index had a higher risk for PE.

Maternal T lymphocytes play an important role in immune response and keep a transient state of tolerance for paternal alloantigens [30]. It has been demonstrated that Treg cell participates in maintaining homeostasis and preventing maternal immune self-reactivity during normal pregnancy [31]. Loss-of-function mutations of the FOXP3 gene can conduce to the functional deficiency of Treg cells in animal and human models [32], which can further inhibit natural killer cells, macrophages, and dendritic cells to affecting the maternal immune tolerance [33]. SNP rs2232365 located in a putative binding site for the transcription factor GATA-3 and its polymorphism was likely to contribute to variant(s) in the quantity or quality of FOXP3 [33]. FOXP3 gene and pregnancy have been extensively studied and proved to be associated with recurrent pregnancy loss in Egyptian [33] and preterm premature rupture in the Zaporizhzhia population [34]. A meta-analysis about the association between the SNP rs2232365 and immune‐related pregnancy complications revealed that allele G and GG or AG genotype were high-risk factors for adverse pregnancy outcomes [35]. In the present study, we identified rs2232365 was associated with a higher risk of LOPE in Northeast women in China and affected the metabolism of lipids.

Immune status is generally correlated with heredity, BMI, lipid metabolism, and nutrition. The level of FOXP3 was markedly elevated in patients with PE who hold abnormal maternal lipids, hyperglycemia, and high BMI [36]. HDL is a vasodilator that interacted with the vascular endothelium and its concentration generally increases throughout the whole pregnancy [37]. HDL carries redundant potentially harmful cholesterol to the liver to excrete reverse cholesterol and protect the maternal vascular endothelium [38]. In the present study, significantly higher levels of serum TG were identified in patients with PE, consistent with previous studies [36]. Therefore, TG/HDL as the risk factor for both EOPE and LOPE, can conveniently reflect the balance between dangerous and protective lipids in patients. We find FOXP3 rs2232365 a novel function of affecting the TG/HDL level in Chinese pregnant women.

FOXO3 rs3761548 was also reported as a risk factor to immune-related pregnancy complications [35] and an important contributor for the progression of PE in Iranian women [39]. While no associations between SNP rs3761548 and preeclampsia were found either in Iranian women [40] or the Turkish population [41]. We thought that the conflicting observations were conduced mainly by ethnic and geographic differences. Mutation of the genotype of rs3761548 mostly affects the expression and activity of FOXP3 protein, which was further involved in many autoimmune diseases including rheumatoid arthritis [42], allergic rhinitis [43], and autoimmune thyroid disease [44]. Our study showed that FOXO3 rs3761548 was not found to be related to preeclampsia in Northeast women of China based on the present date.

Another TLR7 rs3853839 significantly associated with LOPE was found by ϰ^2^ test, which was also related to the levels of FT3. But, the difference did not appear after a logistical regression analysis based on present data. Toll-like receptors (TLRs) which are a family of pattern-recognition receptors promote the activation of autoreactive B cell and elicit innate/adaptive immune responses [45]. Female patients with rs3853839 CC genotype might present a pronounced defensive effect against persistent HCV infection [46] and periodontitis [47]. Otherwise, allele C and SNP rs3853839 are associated with severe hand, foot, and mouth disease (HFMD) [48]. In Chinese women, there was no association between rs3853839 and preeclampsia, and the relationship of FOXP3 gene rs3853839 and thyroid function need further research involving larger samples.

BMI, Thyroid dysfunctions, and dyslipidemia were enrolled in the control-study, and there were significant associations between those parameters and preeclampsia, including EOPE and LOPE subgroups. Pregnancy women who exposed to dyslipidemia are more prone to developing gestational diabetes, preeclampsia, preterm birth, or cardiovascular diseases (CVD) [49, 50]. Thyroid dysfunction, including hypothyroidism and thyrotoxicosis, is associated with preeclampsia, preterm delivery, placental abruptions, and fetal neurologic development [51]. Pre-pregnancy BMI had been identified to be independent risk factors for both EOPE and LOPE, and BMI might be one of the ways to diagnose preeclampsia [52]. Our results are similar to those of previous studies. Changes in thyroid function profiles in women with preeclampsia are controversial in different studies reported. The levels of T3 and T4 hormones are higher in Sudanese patients with preeclampsia [53], but not in Iranians [54]. In our study, we got a consistent and stable result: women with preeclampsia had higher levels of TSH and lower FT3 hormones. The levels of FT3 and FT4 are associated with the tendency of preeclampsia, although the information regarding thyroid function in preeclampsia was scanty [55].

Preeclampsia, known as a complex disease, involves multiple risk factors including genetic susceptibility, immunity, hypothyroidism, and environmental factors. Those predictive values of the traditional screening models of PE were population dependent, basing on maternal medical histories, characteristics, and biophysical and biochemical markers. The Fetal Medicine Foundation (FMF) algorithm had been identified to be super to the method of the National Institute for Health and Care (NICE), and the DRs (95% CI) for EOPE and LOPE were 58.2% (45.5–70.2) and 44.1% (37.3–51.1) respectively [24] To date, no single risk factor as the absolute predictive indicator has been identified. We think that the onset of the disease is based on the cumulative contributions of many risk factors. Pregnant women with high-risk factors should be screened out relying on a predictive model of preeclampsia. According to the individual genetic background of patients, the regulation of their environmental risk maybe a personalized diagnosis and treatment measure worth further study. China is a multi-nationality county with different genetic information, and Chinese Northern Han populations were our targeted populations. In the present study, we identified different risk factors for EOPE and LOPE through the method of traditional logistic regression. Different risk factors are shown in the study, which means there are different pathogenesis in EOPE and LOPE. We think it is a considerable method to predict the risk of preeclampsia based on the cumulative effect of different risk factors. In the further, we will continue to explore the risk factor of preeclampsia to build a model of preeclampsia. Further studies for genetic polymorphism described in maternal-fetal pairs of spontaneous conceptions and oocyte donations will help us to better etiological understanding of PE.

The major limitation of the present study should be noted. That the sample size is relatively small, which will be expanded in both controls, and PE groups with more environmental and genetic factors for optimization and validation of the predictive model.

## Conclusions

A novel function was found that pregnant women with TT/CT genotype of FOXO3 rs2232365 had higher TG/HDL in LOPE. C allele and CC genotype of SNP rs2232365 are associated with the risk of LOPE in Chinse northeast women.

## Data Availability

The datasets used and/or analyzed during the current study are available from the corresponding author on reasonable request.

## Abbreviations

SNP: Single nucleotide polymorphism
FOXO3: Forkhead/winged helix transcription factor
TLR7: Toll-like receptors
Tregs: Regulatory T cells
PE: Preeclampsia
EOPE: Early-onset preeclapmsia
LOPE: Late-onset preeclampsia
BMI: Body mass index
HFMD: Mouth Disease
CVD: Cardiovascular diseases
TSH: Thyroid-stimulating hormone
T3: Triiodothyronine
T4: Thyroxine TC total cholesterol
TG: Triglycerides
HDL: High-density lipoprotein-cholesterol
LDL-C: Low-density lipoprotein-cholesterol
ROC: Receiver Operating Characteristics
CNS: Central nervous system
